# Competence in Sports Pharmacy among Pharmacy Students in Norway

**DOI:** 10.3390/pharmacy12010003

**Published:** 2023-12-23

**Authors:** Natalia Dabrowska, Lone Malmberg, Hadis Nejati, Cecilie Bach Volle, Maren Røssing Witzø, Hatice Yaman, Parisa Gazerani

**Affiliations:** 1 Department of Life Sciences and Health, Faculty of Health Sciences, Oslo Metropolitan University, 0130 Oslo, Norway; 2 Department of Health Science and Technology, Faculty of Medicine, Aalborg University, 9260 Gistrup, Denmark

**Keywords:** sport, pharmacy, sports pharmacy, doping, anti-doping, World Anti-Doping Agency, competence, education

## Abstract

Pharmacists are competent to promote the proper use of medicines. According to the International Pharmaceutical Federation, pharmacists must develop competence in sports pharmacy and the contents of the World Anti-Doping Agency code. This explorative study aimed to identify the status of sports pharmacy in pharmacy education in Norway and competence in sports pharmacy among Norwegian pharmacy students. The study curricula of pharmacy education were examined for the content of sports pharmacy. An online questionnaire was also developed and distributed among pharmacy students. The anonymous survey collected demographic information and data on competence in sports pharmacy. Data from 122 participants were analyzed. Only 22.5% of pharmacy students had acquired a form of training in sports pharmacy and 91.7% wished to gain higher competence. In total, 40.2% of respondents were uncomfortable in advising athletes and trainers on medication use in sports. Study year was found to correlate with competence level with a significant difference between the 3rd year (bachelor) and 5th year (master) students. Age, institution, and number of years engaged in sports were not associated with competence level. The inclusion of sports pharmacy in pharmacy programs holds practical relevance for enhancing competency levels. This implementation can be realized through the integration of sports pharmacy modules and/or the incorporation of research-based activities.

## 1. Introduction

Sport is an integral part of society and possesses considerable social, economic, and political impacts [1]. From a health perspective, sports can provide a large number of benefits to individuals and public health in terms of physical and mental health and overall well-being [2,3]. The negative effects of sports have also been noted in terms of risk of injury, risk of failure in competitions, disturbed mental health and stress, eating disorders, sport-induced gastrointestinal problems, and burnout [4]. These negative impacts are more frequent in elite athletes who are expected to perform at a high level in competitions [5,6,7].

Acquiring higher physical and mental capacity for better performance, and overcoming physical and mental challenges, such as injury and stress, could lead athletes to proper and improper use of medications [8,9,10]. Many drugs [11,12] and dietary supplements [13,14] have been recognized to have performance-enhancing effects and are misused intentionally or unintentionally by athletes [15]. Anabolic agents and stimulants have been among the most frequently consumed agents for performance enhancement, so-called doping [16]. Doping is defined as the use of drugs, substances, or other methods to increase performance by achieving a certain performance-enhancing effect [17]. The World Anti-Doping Agency (WADA) annually provides a prohibited list of banned substances and methods [18]. WADA conducts an annual review and updates the list to include new substances and techniques that become available. Overall, the anti-doping activities and efforts by WADA, in cooperation with national anti-doping agencies, are designed to avoid harm to health and to promote clean and fair sports [18,19]. Pharmacists can play an essential role in promoting the safe and proper use of medications by athletes and can be active players in anti-doping activities [20,21]. The landscape of pharmacists’ roles within the healthcare system has undergone a profound transformation over the last fifty years [22]. Evolving into integral healthcare professionals, pharmacists now play a central role in delivering patient-centered services, championing public health through professional counseling, active participation in healthcare initiatives, and establishing connections with other healthcare practitioners [23]. Their unique position, as accessible figures to the public, involves ensuring the safe and effective administration of pharmaceuticals, engaging in health screening and monitoring initiatives, and being attuned to the preferences and needs of patients. This paradigm shift signifies that pharmacists should extend their activities beyond traditional medicine dispensing, offering additional pharmacy services to comprehensively assess a patient’s overall health and enhance pharmaceutical care outcomes [24]. As the landscape of pharmacy practice evolves, there arises a discernible need for a novel model of pharmacy education [25,26]. Many places around the globe are presently undergoing significant transformations in pharmacy education in response to the evolving landscape of health and healthcare delivery. These reforms necessitate the implementation of robust systems to ensure that the quality of educational structures, processes, and outcomes will generate proficient pharmacy graduates in the years ahead. It is therefore essential for academic pharmacy institutions to establish partnerships with relevant themes, units, and agencies to foster sustainability and achieve positive outcomes. In line with this, identifying and addressing deficiencies in pharmacy curricula is crucial [27].

Since 2014, the International Pharmaceutical Federation (FIP) has outlined pharmacists’ roles in the fight against doping in sports [28]. The guidelines describe expectations on providing sufficient information for the proper use of medicinal products that contain active ingredients listed in the doping list and to inform users about the benefits and risks associated with the use of dietary supplements in sports [28]. Sports pharmacy is a relatively new and emerging line in pharmacy that offers a unique place for pharmacists to acquire sufficient competency to disseminate drug information and guide athletes and trainers [29,30]. Sports pharmacists are, therefore, those pharmacists who are particularly competent in guiding athletes and coaches according to the WADA code and contributing to doping control and clean sport [30]. In addition to education, consultation, and guidance roles to help prevent accidental or intentional doping, sports pharmacists can contribute to the analytical testing of doping control and health administration [20,31,32]. This huge potential seems, however, to remain underdeveloped. In 2022, FIP reported on the limited access to education in sports pharmacy around the world [33] and recommended training in sports pharmacy for all pharmacists [33]. In this report, the importance of institutions and organizations in offering training at basic and advanced levels to establish competence within sports pharmacy was also emphasized [33]. Pharmacists play a crucial frontline role in the dispensation of medications and the provision of advice, extending their expertise to encompass medicines relevant to sports. Given their extensive knowledge of pharmacology and their responsibility in counseling patients, pharmacists are well-positioned to offer support and guidance to athletes concerning the potential risks associated with the inadvertent ingestion of prohibited substances. The prevalence of documented cases involving unintentional use of banned substances, be it through prescribed medications or over-the-counter products, underscores the significant role that pharmacists can fulfill in assisting athletes [32,34].

The literature in this field is limited. A scoping review from 2022 reports on pharmacists’ lack of knowledge and the absence of guidelines for specific roles and responsibilities for pharmacists [32]. This highlights the pivotal role of higher education in the process of building and developing pharmacy students’ competencies, including sports pharmacy. The lack of sufficient training in pharmacy education has been presented by a small number of studies. Redouane et al. [35] reported that pharmacists in Algeria felt undereducated on the subject of sports pharmacy and doping. Awaisu et al. [36] assessed pharmacy students’ knowledge in Qatar and found that 85% of respondents were unaware of the FIP’s statement on the pharmacist’s role in anti-doping, and only half of the students knew the prohibited list of drugs [36]. Shibata et al. [37] demonstrated that most of the students who participated in their study only knew the word doping and had not attended any formal training on the subject, and 41% did not know about over-the-counter medicines and dietary supplements and their potential role as prohibited substances. Lemettila et al. [38] also demonstrated a perception of insufficient knowledge in training sports pharmacy, doping, and anti-doping activities among pharmacists in Finland [38]. Greenbaum et al. [34] found a gap in education among pharmacists in Australia who had some skills to deliver assistance regarding the use of prohibited substances in sports, but many lacked core knowledge and resources to enable them to deliver comprehensive care to prevent harm and doping violations [34]. 

In Norway, six educational institutions offer pharmacy education. It is, however, unknown what the overall status of competence in sports pharmacy and doping is among pharmacy students in Norway and what study plans are currently offered for sports pharmacy and doping in pharmacy education. We conducted this study to identify potential gaps from two aspects: (1) anonymous self-report perceptions from pharmacy students about their competence in sports pharmacy and doping, and (2) the study curricula content of current Norwegian pharmacy programs for training subjects relevant to sports pharmacy, doping, and anti-doping. We anticipated that the findings from this study would raise awareness and facilitate future planning for pharmacy education within the field of sports pharmacy.

## 2. Materials and Methods

### 2.1. Content of Sports Pharmacy in the Study Curricula of Pharmacy Education at Norwegian Institutions

First, we searched the institutions’ official web pages to identify pharmacy programs offered at Norwegian institutions. Only the current study curricula were collected for review to identify the content of programs for training on subjects relevant to sports pharmacy, doping, and anti-doping. Requests were also sent out to the study leaders at the educational institutions to verify our findings via the search. The institutions consisted of the Nord University (offering a 3-year bachelor’s in pharmacy), Oslo Metropolitan University (offering a 3-year bachelor’s and a 2-year master’s), The Arctic University of Norway (offering a 3-year bachelor’s and a 2-year master’s), the Norwegian University of Science and Technology (offering a 2-year master’s), the University of Oslo (offering an integrated 5-year master’s), and the University of Bergen (offering an integrated 5-year master’s). 

### 2.2. Competence in Sports Pharmacy as Perceived by Pharmacy Students at the Norwegian Institutions

#### 2.2.1. Study Design and Target Population

A cross-sectional study was designed to collect information about competence in sports pharmacy from pharmacy students at the six universities that offer pharmacy education in Norway. The target population was defined as pharmacy students in both bachelor’s and master’s programs, with no limitation on age, gender, and location in Norway. The questionnaire was digital, in the Norwegian language, and completely anonymous, hence voluntary to participate in. The cross-sectional study design [39] allowed the collection of large amounts of data in a limited time from the target population. Due to the nature of this study, no ethical approval from the Regional Committee for Medical and Health Research Ethics was required. No additional informed consent was obtained from the participants. It was already announced that through voluntary, anonymous participation, consent is given to analyze and use the data for academic purposes. In addition, the topics covered by the study did not include any personally sensitive data, and therefore approval from the Norwegian data protection agency was not required. The data were collected during April 2023. 

#### 2.2.2. Questionnaire

Since no standard questionnaire was available for the purposes of this study, a survey was created to collect demographic information about pharmacy students and their perception of competence in sports pharmacy. To design the questionnaire, inspiration was obtained from relevant available studies in the literature [35,36,37,38], although none of those could be followed entirely. We attempted to purposefully design the questionnaires to obtain information that could answer our research questions. The questionnaire [40] was prepared in a semi-structured format to contain both open and closed questions. The closed questions were formulated as precoded questions, where the answer options were already given, and the respondent could select an option that was the best fit.

The survey started with a short introduction of the research group, the study purpose, and information about the questionnaire, which consisted of 3 sections. The first section targeted participants’ backgrounds, with questions about basic demographic information such as age, gender, institution, study year, and personal sports experience. The second part focused on the pharmacy students’ self-assessment of their competence level within sports pharmacy. The last part dealt with the status of training in sports pharmacy, to explore how students acquire competence within this topic. Training status was checked by several questions, including an open box option to allow participants to add extra information. 

The final questionnaire consisted of a total of 30 questions (Supplementary Materials). Four of these were follow-up questions that could be filled in only if the answer was yes to the previous question. Six of the questions were qualitative and were designed to collect openly formulated answers from the participants. It must be mentioned that a pilot test was conducted, where a small group completed an earlier version of the questionnaire and provided feedback. The group for the pilot test consisted of 14 students among acquaintances of the study team with (n = 10) and without (n = 4) a pharmacy background. The purpose of the piloting was to ensure the structure, form, and formulation of questions and answers were appropriate and easy to follow and understand. In addition, the time, on average, required for answering the survey was also determined and used for the information section of the main questionnaire. The feedback from the pilot test resulted in a few changes such as the rewording of some questions and some answer options. 

#### 2.2.3. Data Collection and Handling

The final questionnaire was distributed through the online survey tool of the University of Oslo (Netskjema). It was also published on social media (e.g., Facebook) and the university learning platform, Canvas. We expected to reach a minimum of 100 participants [41]. The survey remained open for a few days (18 to 26 April 2023), and raw data from the responses were extracted into Microsoft Excel. The data were then organized and presented using descriptive statistics. We also defined a competence score by considering point intervals of the obtained points (Table 1). This was merely used to give an overall perception of the current competence of the students. 

### 2.3. Statistical Analysis

First, the normal distribution of data was tested graphically with the aid of mean, median, and mode presentation, and according to the distribution, parametric or non-parametric tests were used. The dependent and independent variables and hypotheses that were tested are summarized in Table 2. The significance level (α) was set at 0.05 (5%). If the *p* value was lower than the significance level, the null hypothesis could be rejected, and it could be concluded that there was a correlation between the variables. Several analyses were carried out on the same data material, which could increase the probability of a type 1 error. To reduce the probability of a type 1 error, a Bonferroni correction was applied [42], and an adjusted significance level was used. Confidence intervals of 95% were calculated wherever appropriate for testing hypotheses. The XLMiner Analysis ToolPak extension to the Excel program was used to perform the statistical analyses. 

## 3. Results

### 3.1. The Contents of the Study Curricula in Current Pharmacy Education at Norwegian Institutions

Our search of the study curricula of the six Norwegian universities offering pharmacy education showed very limited content and training aimed directly at sports pharmacy and anti-doping. The University of Oslo was the only institution offering a specific course on this subject, “FARM5140—Sports Pharmacy and Anti-doping” [43]. This course is an elective master’s-level course with 10 study point credits that offers an introduction to the use of medicines for athletes and its relation to the anti-doping rules. The focus is on the role of pharmacists in avoiding doping violations as a result of either intended or unintended doping. The course consists of 17 lectures, 13 student-active teaching lessons (case discussions, quizzes), and participation as an observer in a simulated doping control exercise [43]. The learning objectives of the course as described on the course homepage [43] are presented in Table 3. 

None of the other pharmacy degrees offered a similar course/content in their study curricula when the updated online curricula were examined [44,45,46,47,48,49,50,51]. 

We also contacted the study leaders at these institutions regarding their subject plans. Oslo Metropolitan University and the Norwegian University of Science and Technology stated that they had no specific teaching in sports pharmacy. Oslo Metropolitan University added that topics such as anti-doping and drugs in sports could be mentioned by lecturers when appropriate in teaching. Nord University informed us that they did not have lessons in the field of drug use by athletes, but that they had general education in the effects and side effects (including doping) of drug groups that are potentially used by athletes. The University of Oslo stated that teaching was delivered on the analysis of doping agents from biological sample materials in the compulsory subject “FARM2130—Pharmaceutical analysis”, and that they have an elective course, “FARM5140—Sports Pharmacy and Anti-doping”. 

### 3.2. Findings from the Questionnaire

#### 3.2.1. Demographic Information

In total, 123 individuals responded to the online questionnaire. One was excluded from further analysis due to an incomplete response. The demographic information of the respondents is presented in Table 4. 

#### 3.2.2. Questionnaire Data

Parts 2 and 3 of the questionnaire contained various questions with the answer options of “Yes,” “No,” or “Do not wish to answer”. Table 5 presents data extracted from the questionnaire with these answer options.

Two questions, “Do you know how to check if a substance is prohibited?” and “Have you ever participated in educational activities about doping or sports pharmacy?”, were followed by a free text box where participants could answer in their own words. 

In total, 92 individuals answered the follow-up question “Do you know how to check if a substance is prohibited?” The answers from the participants demonstrated that they would have checked various available resources to them, for instance, 66 (71.7%) indicated that they would have checked the joint catalog (Norwegian “felleskatalogen” for medicine information). Since multiple answers were allowed, we also identified that 29 (31.5%) would have checked Antidoping Norway, 24 (26.1%) would have visited the WADA webpage, and finally, 11 (12.0%) indicated that they could have checked the doping list. Other resources were indicated as medicine searches in the medicines handbook (Norwegian legemiddelhandboka) and the doping list in lovdata (the collection of online legal resources in Norway). Some unspecific answers were “Internet” and “Look it up”.

In total, 22 individuals answered the follow-up question “Have you ever participated in educational activities about doping or sports pharmacy?” Table 6 summarizes the type of teaching about sports pharmacy or doping that the respondents had previously attended.

Of the 122 who answered the question “How comfortable would you feel in advising a trainer or athlete with sports injuries”, 40.2% of respondents (n = 49) answered “uncomfortable,” and only 12.3% (n = 15) were “comfortable”. 

In response to the question, “Which professions/occupations do you think have the most expertise in sports medicine?”, 122 participants answered, with a total collection of 382 answers. A total of 93.4% of the respondents answered that sports doctors/team doctors have the most competence in sports medicine, while 76.2% answered physiotherapists and only 10.7% indicated pharmacists. The rest of the answers consisted of dentists (0), nurses (2.5%), osteopath manual therapists (9.0%), general physicians (10.7%), chiropractors (28.7%), trainers (32.8%), and athletes (49.2%). 

In response to the question, “What jobs can you imagine pharmacists could have as sports pharmacist?”, 365 answers were collected, where work at international/national doping agencies formed 91.8% of responses. The rest of the answers consisted of community pharmacy jobs (36.9%), academic jobs at colleges and universities (48.4%), jobs at sports unions, sports organizations, and sports teams (58.2%), and pharmaceutical companies developing medicines and nutritional supplements or sports nutrition for athletes (63.9%). In the free text option, a few suggestions were added: pharmacists privately work for sports stars, give lectures for sports teams, take part in court cases regarding doping, and work in FIFA (International Association of Football Federation). 

Of the 120 who answered the question “Which of the alternatives do you think would be best to acquire knowledge of sports pharmacy?”, 27.5% wished for a focused topic within an existing course, 21.7% selected an optional course within pharmacy education, 17.5% an individual lecture or a course, and 10.8% suggested a compulsory course within pharmacy education. In total, 5.8% of the respondents wished for “a further education in master’s degree or Ph.D.”, and 16.7% asked for “an online course, E-learning, self-learning courses, webinars within sports pharmacy”. None of the respondents chose the option “doesn’t need more training in it.”

Six participants responded in free text with proposals, which dealt with an optional topic within pharmacy education, information evenings on topics through relevant associations, and more focus on sports pharmacy through social media and as a topic in community pharmacy and pharmacology.

##### Analysis of the Answers to the Specified “Competence” Questions

**Figure 1:**
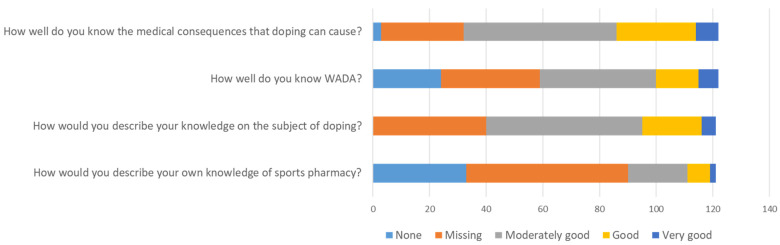
Self-reported competence within sports pharmacy and doping.

In the questionnaires, four particular questions asked participants to self-rate their competence in terms of sports pharmacy and doping. These questions could be answered with a rating from 1 (none) to 5 (very good). Figure 1 presents the outcome of the competence rating. 

The overall competence score was determined as 2.7 out of 5 among the participants of this study, and according to categories defined earlier (see Section 2, Table 1), this represents a moderately good level. Sub-analysis presented that by the graduation year of the bachelor’s courses (the 3rd year), the competence score was 2.60, and by the graduation year of master’s courses (the 5th year), it was 2.89. 

##### Comparisons and Correlational Analysis

The normality test revealed that the distribution of data was normal. Hence, parametric tests were applied. 

**Figure 2:**
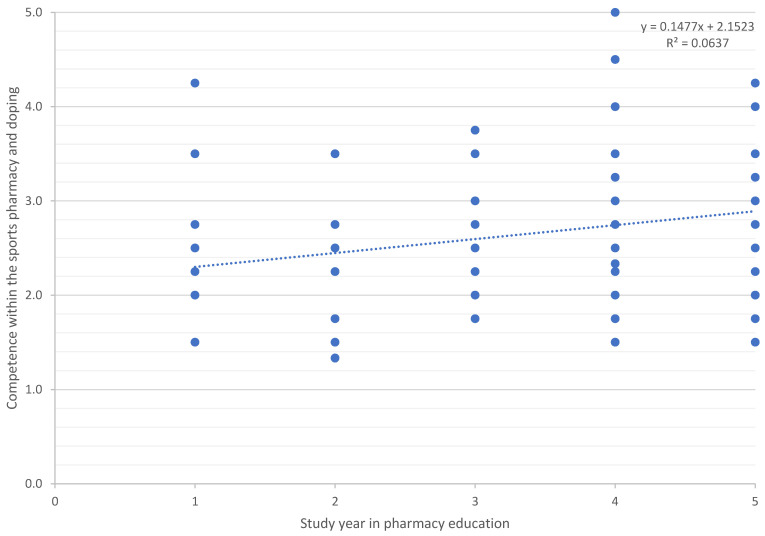
The relationship between study year and competence within the sports pharmacy and doping. *Dots represent data points in the simple regression model*.

Linear regression showed an association between the study year of pharmacy education and competence in terms of sports pharmacy and doping (Figure 2). The R² value for this simple linear regression was 0.06, which can be considered a medium effect size, indicating that the independent variable (study year on the *X*-axis) explains a moderate proportion of the variation in the dependent variable (competence level on the *Y*-axis). 

Whether there was a significant difference between study year 3 and study year 5 and competence was tested, and the result showed a significant difference between these two graduation years. There was no significant difference between the different age groups in terms of their competence. There was also no significant correlation between competence and the number of years one has been engaged in sports. However, there was a significant correlation between competence and whether or not one has played sports. 

No significant difference was found between the educational institutions in terms of the competence level of the pharmacy students. 

Table 7 depicts a summary of the results testing the hypotheses presented earlier in the Section 2. Please note that the Bonferroni correction to adjust the *p*-value yielded *p* < 0.0083 for a result to be statistically significant.

## 4. Discussion

We aimed to assess the state of competence in sports pharmacy and doping among pharmacy students in Norway. Despite a literature search yielding no relevant articles in Norway, we found limited publications globally, including studies from Finland, Qatar, Algeria, and Australia [35,36,37,38]. The Finnish study [38] was considered the most comparable due to similar geographical, economic, and social conditions. However, differences in study populations, target groups, and focus areas precluded direct comparisons. Our study, focusing on pharmacy students, shared similarities with the Qatar study [36], which we used for comparison. Our investigation revealed limited access to sports pharmacy education in Norway and other studied locations [34,35,36,37,38]. The overall insufficient knowledge among pharmacists and pharmacy students worldwide highlights the need for improvement. In the subsequent discussion, we present our study’s findings and, when possible, contrast them with related research.

### 4.1. The Content of Study Curricula at Norwegian Institutions

We searched the websites of the Norwegian institutions that offer pharmacy education to identify the most current study plans and if there is any content related to sports pharmacy, doping, and anti-doping. Officially, only one course was identified at the University of Oslo. This was confirmed by contact with study leaders of pharmacy programs. However, it is worth mentioning that a lecture, a teaching session, or similar activity might have been conducted, without being officially listed in the study plans or as a learning objective of a defined course. The elective course FARM5140—Sports Pharmacy and Anti-doping, at the University of Oslo, has been offered since 2021, and we identified one respondent among our study population who had taken this particular course. As seen in Table 3, the learning objectives of this course include both aspects of general sports pharmacy and analytical aspects for anti-doping activities. Since this course is only offered at the master’s level, we can conclude that the possibility is open for designing and offering more educational content in the study curricula both at the bachelor’s and master’s levels at Norwegian institutions. 

Our findings show a wide range of suggestions on how to acquire knowledge and competence in sports pharmacy. According to our respondents, this can be in the form of a focused topic within an existing course, an optional course within pharmacy education, an individual lecture, a compulsory course within pharmacy education, a continual educational course as part of a master’s degree or Ph.D., an online course in the form of E -learning, a self-learning course, or even educational webinars on sports pharmacy. In fact, with an emerging move toward offering micro-courses or microlearning, one can imagine that sports pharmacy can also be offered in that manner. Micro-credentials [52] are becoming popular, and the European approach to micro-credentials has also been initiated since June 2022 [53] within the concept of lifelong learning. Skills-based hiring and promotion have also gained momentum to put the focus on a person’s skills and competencies. This approach has opened up huge benefits for applicants and employers to match the required specifications and upskill where it is needed.

### 4.2. Competence in Sports Pharmacy, Doping, and Anti-Doping among Pharmacy Students in Norway 

A total of 123 individuals answered the survey, and 122 answers were analyzed. Even though a larger sample size would have been desirable [54], the study population is fairly representative, as we had students from all six institutes. We do not cancel the very low risk of participation from another field of education, but there was no way to control for this in the study design to ensure that strictly pharmacy students could take part. This would have introduced some violations of the anonymity that we wished to maintain. 

We identified a higher number of female (80.3%) than male (19.7%) participants, which potentially originated from the predominance of female students in pharmacy education in Norway. According to farmatid.no, in 2026, 7 out of 10 pharmacy students were females. The reason for this gender distribution in Norway remains unknown; however, a study in 2018 [55] identified that this might be related to job satisfaction, which is lower in men compared with women in pharmacy. 

The age range of our participants was expected, considering the popular studentship age and bachelor and master programs. Predominantly, the respondents were in the age range of 22–25 years old, which accounted for 52.5% of the total number of responses. There was no influence of age on competence, according to our results. We did not test the effect of gender in this regard. 

Our findings show that 95.1% of the participants had heard about cases of doping in sports and consequently could gain a general level of knowledge about doping in sports. This is perhaps reflected by the extensive media coverage of doping cases involving athletes [56]. Traditionally, the Norwegian attitude towards doping has been focused on prevention and providing education to Norwegian athletes [57]. According to Gilberg et al. [57], Norway is one of the leading nations in providing anti-doping information at the public and athletic levels that could have a successful impact on society as a whole. Therefore, a part of the students’ awareness might have been linked to this traditional anti-doping attitude in Norway. 

In total, 76.7% of the respondents stated that they know how to check whether a substance is prohibited in sports, but only 32.0% know what sports pharmacy entails. These results show that although the majority of the pharmacy students in our study population do not know what sports pharmacy is, most are familiar with various resources where they can find the information. This capacity might be rooted in the study curriculum of pharmacy students, where they gain knowledge and training for using trustworthy medication resources, and hands-on skills during pharmacy practice both in the bachelor’s and master’s pharmacy programs. Therefore, it is expected that in terms of the safe use of medications, this group of students is highly competent. 

Only 22.5% of the participants had taken part in teaching related to sports or doping. This finding indicates that some sort of training has been available to participants, which is in line with what has been recommended by the FIP [28,33]. Although the proportion was small, 96.6% of the participants indicated that it is important for pharmacists to know about sports medicine, and 91.7% reported that they are willing to learn through self-education or training in sports pharmacy even if the official academic education might be limited. These findings clearly show the self-awareness and positive attitudes of pharmacy students toward learning and building up competency within sports pharmacy. A similar perception has been reported by previous studies [36,38]. A recent study [34] in Australia, with the aid of a simulated-patient design, examined if pharmacists could give appropriate advice for the use of medication to athletes and found that 68% could give appropriate anti-doping advice and only 11% could give comprehensive clinical and anti-doping advice [34]. The researchers speculated that this might be due to insufficient education in sport-related pharmacy [34]. They also determined that pharmacists who participated in their study were willing to assist athletes, even with insufficient knowledge [34], a trend that has also been observed in other studies [21,58,59]. Therefore, the internal motivation and positive attitudes of pharmacists can be boosted by providing sufficient training to empower them for proper consultation as sports pharmacists. 

Interestingly, we found that when it comes to sports injuries, only 40% of the respondents had some knowledge of treatment options. This result was in line with the level of comfort for advising athletes and trainers in case of sports injury. In response to the level of comfort, 0.8% of respondents felt “very comfortable” advising a trainer or athlete in case of sports injuries, while 40.2% were “uncomfortable”. This reflects that even though pharmacists are trained in guiding the correct use of medicines for patients with various injuries and conditions, and are among the more highly accessible healthcare professionals [60], sports injuries remain a less comfortable area for pharmacists to engage with. This can be eventually potentiated by adding to the learning objectives of study curricula in pharmacy education. 

According to 93.4% of our respondents, sports doctors/team doctors are the most competent in sports medicine at present. Physiotherapists were named as holding second place (76.2%). These results might be based on traditional models for handling sports injuries, where pharmacists are not usually members of the team. Commonly, physiotherapists and occupational therapists are associated with the treatment of injuries and post-injury training. Only 10.7% of respondents selected pharmacists, which gives the impression that either the response is a consequence of low competence, discomfort level, or both. 

It is worth mentioning that other studies have presented a difference between pharmacists and other health professionals. For example, in a study in 2011 conducted in Slovenia, 77% of general practitioners considered themselves poorly prepared and 86% had a lack of sufficient knowledge or training about doping [61]. Another study in 2020, conducted in Pakistan, identified that 65% of physiotherapists had a lack of knowledge about prohibited substances [62]. 

Our results show that respondents could see a potential role in their profession as sports pharmacists [63]. Over 90% of respondents considered such a role in national and international doping agencies as a possible future workplace. Pharmacists’ interdisciplinary knowledge in medication use, pharmacology, medicinal chemistry, and drug analysis are aligned with capabilities desirable for anti-doping activities, and future sports pharmacists in such positions can help to avoid both intentional and unintentional doping [29,30,63,64]. Other opportunities were mentioned as being involved in the development of medicines and dietary supplements specifically targeting athletes, having jobs in various sports associations or associations, and academic careers education. The vision of the current students in our study emphasizes the potential for further development of specialized expertise within pharmacy education and professional pharmacy-related occupations. 

Collectively, the above-mentioned findings highlight a gap in competence that can be filled by offering educational resources and training. When we asked our participants how to acquire sufficient competence in sports pharmacy, 77.5% responded that more focus on sports pharmacy through pharmacy education would be an option. Only 5.8% responded with an option of further education, as a master’s degree or Ph.D. This shows a desire for early engagement in competence building, i.e., in the bachelor program, if possible. They also considered self-training, online courses, e-learning, self-learning courses, and webinars (16.7%), which reflects on their self-esteem and desire for extracurricular competence building. FIP emphasizes that institutions should facilitate education and training to establish sufficient competence in sports pharmacy [28,33]. With UiO as the only university in Norway that offers an elective course in sports pharmacy, there is a need for extended offers within the subject in Norway. Interestingly, the 2021 study in Finland [38] among already employed pharmacists also showed a need for further training in sports pharmacy, doping, and anti-doping. Therefore, it seems that both a basic and continuing education in sports pharmacy might be optimal for acquiring a competence level that empowers pharmacists, according to FIPs. Identification of the most practical design for embedding the subject in a 3- or 5-year program needs further investigation. It is, however, recommended that educational programs such as those provided by Japan’s Anti-Doping Agency (JADA, play true sports pharmacists 2020) [65], or certification programs and online courses offered by WADA would be beneficial to provide pharmacists with appropriate training [66].

We identified a positive linear relationship between competence level and the study year in pharmacy education. We also found a significant difference in competence between the 3rd and 5th study years the years of completing a bachelor’s or a master’s degrees, respectively. This can be grounded in several reasons, for example, a result of progress in general pharmacy competence over the course of education, interest in various job possibilities after graduation, and possibilities to acquire more extracurricular information, for example, through thematic evenings, attending an external talk, or various relevant webinars. However, due to the nature of this study, it was not possible to identify the exact reason.

The average competence score for the participants in the survey was found to be at the “moderately good” level. According to the FIP [33], pharmacists should have a high level of knowledge of sports pharmacy. Hence, we consider a need for additional education in sports pharmacy and eventually integration into pharmacy practice to elevate the competence of and empower pharmacists for their expected roles in relation to sports pharmacy. 

### 4.3. Study Strengths and Limitations 

Our survey was designed as a cross-sectional study, which is appropriate for collecting large amounts of information in a short time, but the downside is missing the development or progress in a longitudinal manner or through a follow-up. To identify the current status, this method was an optimal choice to fulfill our aim. Cross-sectional studies can, however, be designed in various forms; for example, focused group interviews for further in-depth exploration of the topic. We selected an online survey to reach a larger number of participants from various locations in Norway. This method was less complicated, anonymous, and participation was free of charge. Anonymity was assumed to enhance confident participation and honest and direct answers. Easy distribution and collection of data within a short period were the positives, while we could have missed comprehensive answers and clarifications from participants due to the nature of our semi-structured survey. Therefore, it will be highly beneficial to conduct follow-up studies to test whether offering training in sports pharmacy can actually result in higher competence. Such studies can be designed to incorporate a digital interview to provide more detailed answers from the participants. 

The study size is considered a point of limitation, and larger studies are required to enhance the statistical power and reveal whether the findings can be substantiated. We included 122 responses in our analysis, and this is close to the minimum recommended sample for a questionnaire with 30 questions (150). It is commonly accepted that when using a survey instrument to collect data, a minimum of 5 respondents per question is required. In addition, a national-based survey can estimate the target group size and calculate the response rate. Nevertheless, a response rate of 30% is considered acceptable, whereas an excellent response rate is determined as being 50% or higher. We did not have the exact number of our target group and we cannot determine if the questionnaire reached the entire target group in this study. However, based on the number of master of pharmacy students in 2022, we estimated a response rate of 20% in our study. Additionally, the questionnaire itself needs further validation, even though the first two steps of face validity and pilot testing that were used in the validation process were conducted to some extent, principal components analysis was lacking. We also consider the risk of recall bias in our study. In addition, we cannot rule out whether voluntary participation was based on a neutral responder type or an interested/engaged responder who might have already been interested in/know about sports pharmacy, doping, and anti-doping activities. Our results showed that having played sports was associated with sports pharmacy competence but the number of years of being engaged in sports did not show any association. 

This study demonstrates existing gaps and forthcoming opportunities, emphasizing the pragmatic significance of incorporating educational modules and research-based initiatives in sports pharmacy within pharmacy programs. Pharmacists, esteemed as specialists offering pharmaceutical care, emerge as pivotal figures in supporting athletes and sports personnel in the prevention of doping. Furthermore, the study shows the pivotal role of meticulously selecting materials and topics directly pertinent to pharmacy practice when planning future education for pharmacy professionals. For community pharmacists, enhancing competency in diverse facets of sports pharmacy, such as the identification of prohibited substances, provision of pharmaceutical care for athletes, management of therapeutic use exemptions, and oversight of doping outcomes, is deemed imperative. As the field of sports pharmacy garners increased recognition globally [67], delving into the educational content for professional advancement draws a roadmap for future research endeavors.

## 5. Conclusions

The proficiency of Norwegian pharmacy students in sports pharmacy has been determined to be moderately satisfactory. However, the revelation that only 22.5% of pharmacy students have received formal academic training in sports pharmacy highlights the need for further enhancements. Our study indicates a correlation between the level of competence and the study year, with 91.7% of the participating pharmacy students expressing a desire to engage in some form of sports pharmacy training.

In light of these findings, we recommend considering integrating basic and advanced levels of sports pharmacy into the curricula of bachelor and master programs in pharmacy. This incorporation can take various forms, including integration into existing courses or the introduction of specialized optional courses. Presently, among the six universities in Norway, UiO is the sole institute offering an elective course in sports pharmacy and doping. Nevertheless, our results reveal no significant differences in the competence levels of students from different institutes. Age and gender do not appear to influence competence levels, but a greater number of study years and active participation in sports are associated with increased proficiency. Considering the overall outcomes, we advocate for the incorporation of sports pharmacy into pharmacy education in Norway.

## Figures and Tables

**Table 1:** Score intervals for the competence score.

Answer Option	Number of Points in Answer (1 to 5)	Score Interval Defined for the Competence
“Very good”	5	≥4.5
“Good”	4	3.5–4.4
“Moderately good”	3	2.5–3.4
“Missing”	2	1.5–2.4
“None”	1	≤1.4

**Table 2:** Overview of null hypotheses, independent variables, dependent variables, and statistical analysis.

Null Hypotheses	Independent Variable	Dependent Variable	Statistical Analysis
H0 = There is no difference in the competence in sports pharmacy between the study years in pharmacy education	Study year	Competence	Linear regression
H0 = There is no difference in competence in sports pharmacy between the 3rd year (bachelor’s) and 5th year (master’s) students	3rd and 5th study year	Competence	Independent *t*-test
H0 = There is no difference in competence in sports pharmacy between the educational institutions	Educational institutions	Competence	Analysis of variance (ANOVA)
H0 = There is no relationship between competence in sports pharmacy and age	Age	Competence	Linear regression
H0 = There is no relationship between competence in sports pharmacy and sports activity	Active in sport	Competence	Independent *t*-test
H0 = There is no relationship between competence in sports pharmacy and the number of years of sports activity	Years of sports activity	Competence	Linear regression

**Table 3:** Learning objectives of FARM5140—Sports Pharmacy and Anti-doping, University of Oslo. *It is expected that students, upon completion of the course, acquire knowledge about the presented topics*.

Knowledge about the role and responsibility of medical support personnel related to the administration of drugs to athletes and the role of pharmacists in the prevention of doping;
Knowledge about the anti-doping rules (World Anti-Doping Code, international standards, national rules) and national and international anti-doping work;
Knowledge about the WADA’s prohibited list, therapeutic use exemptions, and the Norwegian drug search database, and how to use this knowledge to advise athletes on the correct use of drugs;
Knowledge about common diagnoses amongst athletes and the treatment thereof, included pain management, anti-inflammatory drugs, asthma treatment, hormonal treatment, and the use of stimulating drugs, including drugs of abuse
Knowledge about doping analysis, including procedures, methods, result management, and the dosing of drugs considering the prohibited list and doping analysis;
Knowledge about the use of dietary supplements, and the use of this knowledge to do risk assessments and prevention of doping both in sport and society.

**Table 4:** Overview of information on the questionnaire’s respondents.

Information	Total Number of Respondents	Response Options	Number of Answers (%)
Gender	122	Female	98 (80.3)
		Male	24 (19.7)
		Other	0 (0)
		Do not wish to respond	0 (0)
Age (years)	122	18–21	22 (18.0)
		22–25	64 (52.5)
		26–29	28 (23.0)
		30–33	4 (3.3)
		34–37	1 (0.8)
		>38	3 (2.5)
Educational institute	122	UiO	21 (17.2)
		UiB	14 (11.5)
		UiT	18 (14.8)
		OsloMet	43 (35.2)
		Nord	15 (12.3)
		NTNU	11 (9.0)
Study year	122	5	33 (27.0)
		4	24 (19.7)
		3	39 (32.0)
		2	13 (10.7)
		1	13 (10.7)
How often do you follow sports?	122	Often	23 (18.9)
		Sometimes	70 (57.4)
		Never	28 (23.0)
		Do not wish to respond	1 (0.8)
Have you been involved in any form of sport/sports? (has two follow-up questions)	122	Yes	89 (73.0)
		No	32 (26.2)
		Do not wish to respond	1 (0.8)
If yes: How long did you/have you been involved in sport/sports activities? (years)	89	<1	1 (1.1)
		1–3	16 (18)
		4–6	19 (21.3)
		7–9	16 (18)
		10–12	15 (16.9)
		13–15	10 (11.2)
		>15	9 (10.1)
		Do not wish to respond	3 (3.4)
If yes: What is the highest level you have engaged in sports/sports activities?	89	International level	4 (4.5)
		National level	8 (9.0)
		Regional level	29 (32.6)
		Leisure sports/sports	48 (53.9)
		Do not wish to respond	0 (0.0)

Nord (Nord University), NTNU (Norwegian University of Science and Technology), OsloMet (Oslo Metropolitan University), UiB (University of Bergen), UiO (University of Oslo), UiT (The Arctic University of Norway).

**Table 5:** Overview of answers to sports pharmacy-related questions.

Question	Total Number of Respondents	Number of “Yes” (%)	Number of “No” (%)	“Do Not Wish to Answer”
Do you know what the term sports pharmacy implies?	122	39 (32.0)	83 (68.0)	0
Do you know how to check if a substance is prohibited?	122	92 (76.7)	28 (23.3)	2
Have you heard about some of the previous cases of sports doping (e.g., in news, articles)?	122	116 (95.1)	6 (4.9)	0
Do you know about the treatment of acute sports injuries?	122	48 (40.0)	72 (60.0)	2
Do you think it is important that pharmacists have knowledge of sports medicine?	122	115 (96.6)	4 (3.4)	3
Have you ever participated in educational activities about doping or sports pharmacy?	122	27 (22.5)	93 (77.5)	2
Would you like to attend a training activity about sports pharmacy?	122	110 (91.7)	10 (8.3)	2

**Table 6:** Type of teaching about doping or sports pharmacy that the respondents have attended.

Type of Teaching about Doping or Sports Pharmacy	Number of Answers
FARM5140—Sports pharmacy and anti-doping subject at UiO	6
A lecture/seminar at the university	4
Teaching in analysis—doping tests	3
Lecture given by Antidoping Norway and/or other organizations	2
Teaching at university	1
A teaching day at the university	1
In connection with sport	1
Online course (Anti-doping)	1
Teaching at junior high school and/or upper secondary school	1
Anti-doping	1
“Learned about WADA and that athletes/others can order confirmation of doping search for drugs they use”	1

**Table 7:** Results from testing the study hypotheses.

Hypothesis	Statistical Analysis	*p*-Value	H0: Rejected/Inconclusive (With Bonferroni Correction)
H0 = There is no difference in the competence in sports pharmacy between the study years in pharmacy education	Linear Regression	0.005	Rejected
H0 = There is no difference in competence in sports pharmacy between the 3rd year (bachelor’s) and 5th year (master’s)	Independent *t*-test	1.20 × 10^−13^	Rejected
H0 = There is no difference in competence in sports pharmacy between the educational institutions	Analysis of variance	0.119	Inconclusive
H0 = There is no relationship between competence in sports pharmacy and age	Linear regression	0.962	Inconclusive
H0 = There is no relationship between competence in sports pharmacy and sports activity	Independent *t*-test	5.41 × 10^−43^	Rejected
H0 = There is no relationship between competence in sports pharmacy and the number of years of sport activity	Linear regression	0.034	Inconclusive

Note: Inconclusive means *p*-value > Bonferroni correction (0.0083).

## Data Availability

Data are contained within the article and Supplementary Materials.

## Supplementary Materials

The following supporting information can be downloaded at: https://www.mdpi.com/article/10.3390/pharmacy12010003/s1, The supplementary material is the original Norwegian questionnaire and its translated version in English.
