# Supplementary material for: Competence in Sports Pharmacy among Pharmacy Students in Norway
Source: Pharmacy (Basel). 2023 Dec 23;12(1):3. doi: 10.3390/pharmacy12010003 (PMC10801607; doi:10.3390/pharmacy12010003)
Supplement: Supplementary file 1 [file pharmacy-12-00003-s001.zip › pharmacy-2673264-supplementary.pdf]

Hva er status på kompetansen innen sportsfarmasi hos studenter i farmasiutdanningen i Norge? kopi

Hva er status på kompetansen innen sportsfarmasi hos studenter i farmasiutdanningen i Norge?

Hei!
Vi er en gruppe på 6 farmasistudenter som nå er i gang med å skrive vår bacheloroppgave. I denne sammenheng sender vi deg nå en kort spørreundersøkelse. Målet med studien vår er å undersøke status på kompetansen innen sportsfarmasi hos studenter i farmasiutdanningen i Norge.

Spørreundersøkelsen består av 3 deler, som henholdsvis handler om bakgrunnsinformasjon, kunnskap om sportsfarmasi og alternativer for å forbedre kunnskapen rundt sportsfarmasi. Alle svar registreres helt anonymt, så kom gjerne med ærlige tilbakemeldinger. Undersøkelsen skal svares fra egen erfaring og kunnskap, uten hjelpemidler.

Det tar ca 5-8 minutter å fullføre spørreundersøkelsen, og din tilbakemelding vil være til stor hjelp for oss i vårt arbeid. Spørreundersøkelsen stenges 26.april klokken 12:00.

På forhånd takk!
Med vennlig hilsen,
Natalia Dabrowska, Lone Malmberg, Hadis Nejati, Cecilie Bach Volle, Maren Røssing Witzø og Hatice Yaman

DEL 1: Bakgrunnsinformasjon om deltager

1. Kjønn

- Mann
- Kvinne
- Annet
- Ønsker ikke svare

2. Alder (år)

- 18-21
- 22-25
- 26-29
- 30-33
- 34-37
- >38

3. Utdanningsinstitusjon

- Universitet i Tromsø
- OsloMet - storbyuniversitet
- Norges teknisk-naturvitenskapelig universitet
- Universitet i Bergen
- Nord universitetet

Universitet i Oslo

4. Nåværende årstrinn i utdanningen

1.
2.
3.
4.
5.

5. Hvor ofte følger du med på idrett/sport?

Ofte
I blant
Aldri
Ønsker ikke å svare

6. Har du drevet med/driver med noe form for idrett/sport?

Ja
Nei
Ønsker ikke å svare

7. Hvor lenge drev du/har du drevet med idrett/sport?

Dette elementet vises kun dersom alternativet «Ja» er valgt i spørsmålet «6. Har du drevet med/driver med noe form for idrett/sport?»

<1 år
1-3 år
4-6 år
7-9 år
10-12 år
13-15 år
>15 år
Ønsker ikke å svare

8. Hva er det høyeste nivået du har drevet med idrett/sport på?

Dette elementet vises kun dersom alternativet «Ja» er valgt i spørsmålet «6. Har du drevet med/driver med noe form for idrett/sport?»

Internasjonalt nivå
Nasjonalt nivå
Regionalt nivå
Fritid sport/idrett
Ønsker ikke å svare

DEL 2: Hva kan du innen temaet?

I denne delen er det samlet en del kunnskapsspørsmål innen temaet sportsfarmasi. Vi vil vite hva du kan, derfor skal delen gjennomføres uten hjelpemidler!
Svaralternativ varierer mellom ja/nei, skala fra 1-5 og flervalgsspørsmål.

9. Vet du hva begrepet sportsfarmasi innebærer?

Ja

Nei
Ønsker ikke svare

10. Hvordan ville du beskrevet din egen kunnskap om sportsfarmasi?

5 - Svært god
4 - God
3 - Middels god
2 - Manglende
1- Ingen
Ønsker ikke å svare

11. Hvordan vil du beskrive din kunnskap innen temaet doping?

5 - Svært god
4 - God
3 - Middels god
2 - Manglende
1- Ingen
Ønsker ikke å svare

12. Hvor god kjennskap har du til WADA?

5 - Svært god
4 - God
3 - Middels god
2 - Manglende
1- Ingen
Ønsker ikke å svare

13. Hvor god kjennskap har du til medisinske konsekvenser som doping kan føre til?

5 - Svært god
4 - God
3 - Middels god
2 - Manglende
1- Ingen
Ønsker ikke å svare

14. Vet du hvordan du kan sjekke om et stoff er forbudt i idretten?

Ja
Nei
Ønsker ikke å svare

15. Hvis ja; hvordan ville du selv sjekket dette?

Dette elementet vises kun dersom alternativet «Ja» er valgt i spørsmålet «14. Vet du hvordan du kan sjekke om et stoff er forbudt i idretten? »

16. Har du hørt om noen tidligere saker om doping i idretten? (f.eks nyhetsartikler)

Ja
Nei
Ønsker ikke å svare

17. Har du kjennskap til behandling av akutte idrettsskader?

Ja
Nei
Ønsker ikke å svare

18. Hvor komfortabel ville du følt deg med å gi råd til en trener eller idrettsutøver ved idrettsskader?

5 - Svært komfortabel
4 - Komfortabel
3 - Verken eller
2 - Ukomfortabel
1 - Svært ukomfortabel
Ønsker ikke å svare

19. I hvilke/hvilket av kroppens organsystemer/deler tror du de fleste idrettsskader oppstår?

Det kardiovaskulære systemet
Nervesystemet
Respirasjonssystemet
Muskel- og skjelettsystemet
Vev- og bløtdelsorganer
Fordøyelsessystemet
Ekskresjonssystemet
Det endokrine systemet
Ønsker ikke å svare

20. Synes du det er viktig at farmasøyter har kunnskap innen sportsmedisin?

Ja
Nei
Ønsker ikke å svare

21. Hvilke profesjoner/yrker tror du har mest kompetanse innenfor sportsmedisin NÅ?

Farmasøyter
Fastleger
Fysioterapeuter
Idrettsutøvere
Kiropraktor
Sportsleger/lagleger
Sykepleiere

Tannleger
Trenere
Osteopater
Ønsker ikke å svare

22. Var det noen profesjoner/yrker du savnet i forrige spørsmål?

23. Hvilke jobber kan du se for deg at farmasøyt kunne hatt som sportsfarmasøyt?

Arbeide i utdanning/undervisning på høyskole eller universitet
Jobbe for nasjonale eller internasjonale dopingbyrå
Farmasøytiske selskaper som utvikler medisiner og kosttilskudd for idrettsutøvere
Jobbe i idrettsforbund, -lag eller -foreninger
Jobbe i apotek
Ønsker ikke å svare

24. Har du noen andre forslag til relevante jobber enn alternativ nevnt ovenfor?

DEL 3: Hvordan kan vi forbedre kunnskapen innen sportsfarmasi?

Formålet i denne delen er å utforske hvordan man kan tilegne seg kunnskap innen sportsfarmasi.

25. Har du deltatt i undervisning om doping eller sportsfarmasi tidligere?

Ja
Nei
Ønsker ikke å svare

26. Hva slags undervisning om doping eller sportsfarmasi har du deltatt i tidligere?

Dette elementet vises kun dersom alternativet «Ja» er valgt i spørsmålet «25. Har du deltatt i undervisning om doping eller sportsfarmasi tidligere? »

27. Hvilket av disse alternativene tror du at ville vært best for å tilegne seg kunnskap om sportsfarmasi?

En videreutdannelse (mastegrad/phd)
Et obligatorisk emne på farmasiutdanningen
Et valgfritt emne på farmasiutdanningen
Et fokustema innad i et eksisterende fag
En forelesning/et kurs om det
Nettkurs, E-læring, selv-læringskurs eller webinar
Trenger ikke mer opplæring innen det
Ønsker ikke å svare

28. Er det noen andre tiltak du ville foreslått for å øke kunnskapen om sportsfarmasi/doping?

29. Ville du selv ha ønsket å delta på opplæring om sportsfarmasi?

Ja
Nei
Ønsker ikke å svare

Nettskjema

30. Er det noe annet du vil legge til?

What is the status of competence in sports pharmacy among students in pharmacy education in Norway?

Hello!

We are a group of 6 pharmacy students who are now writing our bachelor thesis. In this regard, we are now sending you a short survey. The aim of our study is to investigate the status of competence in sports pharmacy among students in pharmacy education in Norway.

The survey consists of 3 parts, which respectively deal with background information and knowledge on sports pharmacy and alternatives to improve knowledge around sports pharmacy. All responses are recorded completely anonymous, so feel free to give honest feedback. The survey must be answered from your own experience and knowledge, without aids.

It takes about 5-8 minutes to complete the survey, and your feedback will be of great help us in our work. The survey closes on 26 April at 12:00.

Thanks in advance!

Sincerely,

Natalia Dabrowska, Lone Malmberg, Hadis Nejati, Cecilie Bach Volle, Maren Røssing Witzø and Hatice Yaman

PART 1: Background information about the participant

1. Gender

Man

Woman

Other

Don't want to answer

2. Age (years)

18-21

22-25

26-29

30-33

34-37

38 and above

3. Educational institution

University of Tromsø

OsloMet – Oslo Metropolitan University

The Norwegian University of Science and Technology

University of Bergen

North University

University of Oslo

4. Current year in the education

1.

2.

3.

4.

5.

5. How often do you do sport/sports?

Frequent

Amongst you

Never

Don't want to answer

6. Have you been involved in/are you involved in any kind of sport/sports?

Yes

No

Don't want to answer

7. How long did you/have you been doing sports/sport?

This element is only displayed if the alternative «Yes» is selected in the question «6. Have you been/are you involved in any kind of sports?»

less than 1 year

1-3 years

4-6 years

7-9 years

10-12 years

13-15 years

more than 15 years

Don't want to answer

8. What is the highest level you have played sports at?

This element is only displayed if the alternative «Yes» is selected in the question «6. Have you been/are you involved in any kind of sports?»

International level

National level

Regional level

Leisure sports/sports

Don't want to answer

PART 2: What can you do within the theme?

In this part, a number of knowledge questions within the theme of sports pharmacy have been collected. We want to know what you can, therefore this part must be answered by you without aids!

Answer alternatives vary between yes/no, scale from 1-5 and multiple choice questions.

9. Do you know what the term sports pharmacy entails?

Yes

No

Don't want to answer

10. How would you describe your own knowledge of sports pharmacy?

5 - Very good

4 - Good

3 - Moderately good

2 - Missing

1- None

Don't want to answer

11. How would you describe your knowledge on the subject of doping?

5 - Very good

4 - Good

3 - Moderately good

2 - Missing

1- None

Don't want to answer

12. How well do you know WADA?

5 - Very good

4 - Good

3 - Moderately good

2 - Missing

1- None

Don't want to answer

13. How well do you know the medical consequences that doping can lead to?

5 - Very good

4 - Good

3 - Moderately good

2 - Missing

1- None

Don't want to answer

14. Do you know how you can check if a substance is prohibited in sport?

Yes

No

Don't want to answer

15. If yes; how would you check this yourself?

This element is only displayed if the alternative «Yes» is selected in the question «14. Do you know how you can check if a substance is banned in sports? »

16. Have you heard of any previous cases of doping in sports? (e.g. news articles)

Yes

No

Don't want to answer

17. Do you know how to treat acute sports injuries?

Yes

No

Don't want to answer

18. How comfortable would you feel giving advice to a coach or athlete for sports injuries?

5 - Very comfortable

4 - Comfortable

3 - Neither or

2 - Uncomfortable

1 - Very uncomfortable

Don't want to answer

19. In which of the body's organ systems/parts do you think most sports injuries occur occurs?

The cardiovascular system

The nervous system

The respiratory system

Musculoskeletal system

Tissue and soft tissue organs

The digestive system

The excretory system

The endocrine system

Don't want to answer

20. Do you think it is important that pharmacists have knowledge in sports medicine?

Yes

No

Don't want to answer

21. Which professions/occupations do you think have the most expertise in sports medicine?

Pharmacists

GPs

Physiotherapists

Athletes

Chiropractor

Sports doctors/team doctors

Nurses

Dentists

Trainers

Osteopaths

Don't want to answer

22. Were there any professions you missed in the previous question? ---------

23. What jobs can you imagine a pharmacist could have as a sports pharmacist?

Work in education/teaching at a college or university

Work for national or international doping agencies

Pharmaceutical companies that develop drugs and nutritional supplements for athletes

Work in sports federations, teams or associations

Work in a pharmacy

Don't want to answer

24. Do you have any other suggestions for relevant jobs than the alternative mentioned above? -------

PART 3: How can we improve knowledge in sports pharmacy?

The purpose of this part is to explore how to acquire knowledge in sports pharmacy.

25. Have you attended classes on doping or sports pharmacy before?

Yes

No

Don't want to answer

26. What kind of training on doping or sports pharmacy have you attended in the past?

This element is only displayed if the alternative «Yes» is selected in the question «25. Have you attended classes on doping or sports pharmacy earlier? »

27. Which of these alternatives do you think would be best to acquire knowledge of sports pharmacy?

A further education (master's degree/PhD)

A compulsory subject in pharmacy education

An optional subject in the pharmacy education

A focus theme within an existing subject

A lecture/course about it

Online course, E-learning, self-learning course or webinar

No more training is needed in that area

Don't want to answer

28. Are there any other measures you would suggest to increase knowledge about sports pharmacy/doping?

29. Would you have wished to participate in training on sports pharmacy yourself?

Yes

No

Don't want to answer

30. Is there anything else you would like to add? ---------
